# Refining and testing the diagnostic accuracy of an assessment tool (PAT-POPS) to predict admission and discharge of children and young people who attend an emergency department: protocol for an observational study

**DOI:** 10.1186/s12887-018-1268-7

**Published:** 2018-09-17

**Authors:** Samah Riaz, Andrew Rowland, Steve Woby, Tony Long, Joan Livesley, Sarah Cotterill, Calvin Heal, Damian Roland

**Affiliations:** 1 Clinical Research Unit, Fairfield General Hospital, Bury, UK; 2 Emergency Department, North Manchester General Hospital, Manchester, UK; 3 School of Health & Society, University of Salford, Salford, UK; 4 The Pennine Acute Hospitals NHS Trust, Manchester, UK; 5 Northern Care Alliance NHS Group, Salford, UK; 6 Centre for Biostatistics, University of Manchester, Manchester, UK; 7 SAPHIRE Group, Health Sciences, University of Leicester, Leicester, UK; 8 Paediatric Emergency Medicine Leicester Academic (PEMLA) Group, Children’s Emergency Department, Leicester Royal Infirmary, Leicester, UK

**Keywords:** Paediatric, Emergency department, Diagnostic accuracy, Early identification systems, screening tool, Observational, Early warning score, Early warning system, hospital admissions

## Abstract

**Background:**

Increasing attendances by children (aged 0–16 years) to United Kingdom Emergency Departments (EDs) challenges patient safety within the National Health Service (NHS) with health professionals required to make complex judgements on whether children attending urgent and emergency care services can be sent home safely or require admission. Health regulation bodies have recommended that an early identification systems should be developed to recognise children developing critical illnesses. The Pennine Acute Hospitals NHS Trust Paediatric Observation Priority Score (PAT-POPS) was developed as an ED-specific tool for this purpose. This study aims to revise and improve the existing tool and determine its utility in determining safe admission and discharge decision making.

**Methods/design:**

An observational study to improve diagnostic accuracy using data from children and young people attending the ED and Urgent Care Centre (UCC) at three hospitals over a 12 month period. The data being collected is part of routine practice; therefore opt-out methods of consent will be used. The reference standard is admission or discharge. A revised PAT-POPs scoring tool will be developed using clinically guided logistic regression models to explore which components best predict hospital admission and safe discharge. Suitable cut-points for safe admission and discharge will be established using sensitivity and specificity as judged by an expert consensus meeting. The diagnostic accuracy of the revised tool will be assessed, and it will be compared to the former version of PAT-POPS using ROC analysis.

**Discussion:**

This new predictive tool will aid discharge and admission decision-making in relation to children and young people in hospital urgent and emergency care facilities.

**Trial registration:**

NIHR RfPB Grant: PB-PG-0815-20034.

ClinicalTrials.gov: 213469. Retrospectively registered on 11 April 2018.

## Background

In 2016–2017 4.49 million children aged under 16 years of age attended United Kingdom (UK) Emergency Departments (EDs), up from 4.36 million in the previous year [1, 2]. Current trends continue to demonstrate increasing attendances across a range of conditions [3]. This use of urgent and emergency care facilities puts pressure on the National Health Service (NHS) to balance public demand for high quality services and maintain commissioner and productivity agendas [4]. Ultimately healthcare professionals make judgements on whether children attending EDs can be sent home safely or require admission to a hospital ward or admission to an observation and assessment area. These judgements require a complex assessment of the child’s health and an estimation of the potential for improvement or deterioration. The majority of parents seeking advice for sick children need only reassurance and minimal intervention as there is fortunately a low incidence of serious illness in the UK. However, amongst those presenting each year there are some particularly sick children and young people, and detection requires health care professionals to have skills in recognising them. The National Patient Safety Agency (a predecessor of the NHS Commissioning Board Special Health Authority) and the National Institute for Health and Clinical Excellence supported the conclusions of The Confidential Enquiry into Maternal and Childhood Health (CEMACH) report “Why Children Die: a pilot study (2006)” [5] which highlighted death may be prevented if clinicians were better at recognising deterioration. The report recommended that early identification systems to recognise children developing critical illness should be used as the UK continues to perform poorly against other European countries in relation to childhood mortality [6] calls to introduce these nationally have continued.

A single early warning system is unlikely to perform well across all areas of care, as monitoring a child over a period of time on a hospital ward for the development of worsening illness is different to assessing a child in a relatively short space of time in the ED. There is a need for a specific ED early warning system, validated on ED patients [7, 8].

A recent review of the use of nine paediatric early warning scores in EDs determined they were of only poor-moderate use in the prediction of admission [9]. This study did not examine the safety profile of the scores or whether they could be used to assist in supporting safe discharge decisions. A risk-averse strategy of referring all children of ‘potential concern’ to inpatient paediatric services overloads an already stretched system and leads to unnecessary hospital admissions. These unnecessary admissions are not welcomed by children, families or carers and may cause concern to be expressed by commissioners and financial controllers. There are a limited number of studies on the use of specific scoring systems in children’s EDs and other urgent care settings. For example, a study in 2008 of less than 400 patients demonstrated low sensitivity in predicting the need for admission [10]. The Pennine Acute Hospitals NHS Trust Paediatric Observation Priority Score (PAT-POPS) [11] is a modified version of the Paediatric Observation Priority Score (POPS) [12]. POPS was developed as a bespoke ED specific method of identifying children with potentially serious illnesses or infections while at the same time safely supporting staff in redirecting or discharging those who do not need ongoing immediate care. In other words, sick children can be clearly identified early in the patient journey, and conversely, there is an objective measurement to help staff avoid unnecessary burdening of hospital paediatric services for well children. The initial POPS study demonstrated an increased relative risk of admission with a POPS > 2, and demonstrated the utility of its novel nurse “gut feeling” (judgement) component [13]. Further data on over 20,000 patients has demonstrated a relationship between length of stay and increasing POPS score [14]. POPS has been shown to be beneficial in defining appropriate admission and also effective in defining safe discharge [12–15].

PAT-POPS contains clinical variables includes heart rate, respiratory rate, temperature and also some comments on the appearance of the child (such as work of breathing and level of alertness). Each of the variables is assigned a score between 0 and 2 (i.e. a normal heart rate for the child’s age would score 0; a very high rate would score 2). Nine variables are considered, leading to a score between 0 and 18. Initial study of PAT-POPS showed reasonable sensitivity and specificity of admission prediction (Receiver Operating Characteristic of 0.72 with 95% CI 0.68 to 0.75) compared to other similar tools but probably less than would be clinical acceptable [11]. There is no direct adult equivalent tool as current systems often employ more uncomfortable (e.g. Blood Pressure) or invasive investigations (e.g. blood tests) which would not be suitable in large populations of children [16]. Improving the performance of PAT-POPS could have the following impact in urgent and emergency care settings.

1. Identifying those children and young people that need to be admitted and are more likely to be sicker than those who can be discharged will have beneficial effects on patients as they can be identified more quickly and more reliably, and prioritised for urgent, senior medical care. PAT-POPS ought to improve time to recognition especially in critical conditions like sepsis which can be difficult to recognise.
2. Those children who are sick enough to need inpatient treatment must be able to access it rapidly; conversely well children ought not to be in hospital where they are being taken away from their normal social and family arena. The PAT-POPS tool ought to be able to identify well children and young people that are well enough to be referred back to primary care or self-care at home, as well as to identify those children and young people who require a full assessment and admission. This will not only have beneficial effects on both groups of children but will also lead to service efficiency without jeopardising patient safety.

The overall aim of this project is to revise the PAT-POPS assessment tool to aid discharge and admission decision-making in relation to children and young people in hospital urgent and emergency care facilities, and thereby improve the quality of care that patients receive. The study will examine the feasibility of using PAT-POPS as an assessment tool to estimate the need for hospital admission of children and young people attending EDs and Urgent Care Centres (UCC).

The objectives are:

1. To refine the existing PAT-POPS screening tool, by assessing which combination of components best predicts hospital admission/discharge, and whether the addition of new items can improve its predictive power.
2. To select appropriate cut-points to predict hospital admission and discharge and assess the diagnostic accuracy of PAT-POPS.
3. To validate PAT-POPS by repeating the assessments of diagnostic accuracy in an independent dataset.

## Method

### Study setting

The study will take place in three hospitals at The Pennine Acute Hospitals NHS Trust. They include two general EDs and one Urgent Care Centre.

### Study population

#### Inclusion criteria

Children and young people aged under 16 years of age who attend the ED/UCC at one of the three hospitals.

#### Exclusion criteria

Children and young people who are confirmed dead on arrival at the ED/UCC; children and young people who attend the ED/UCC in cardio-respiratory arrest.

### Outcome measures

The primary outcome measure is admission or discharge.

### Recruitment

The study population will be recruited consecutively and data collection has been planned prospectively. The data being collected from patients is the same as that routinely collected from patients who attend EDs at the current time – it is non-invasive physiological data combined with a subjective assessment of how unwell the patient is. Both of these types of data are routinely collected in EDs throughout the UK and the only difference in this study is that we will capture data on all patients attending during the time period of the study – including those with very minor conditions who, perhaps, would not have had non-invasive physiological measurements taken in some emergency departments. The study will take place over a whole year (February 2018 to January 2019) as inclusion of data from those attending during autumn, winter, summer and spring periods the study will avoid the effects of bias from seasonal variability (e.g. greater incidence of respiratory conditions during the winter months).

**Fig. 1:**
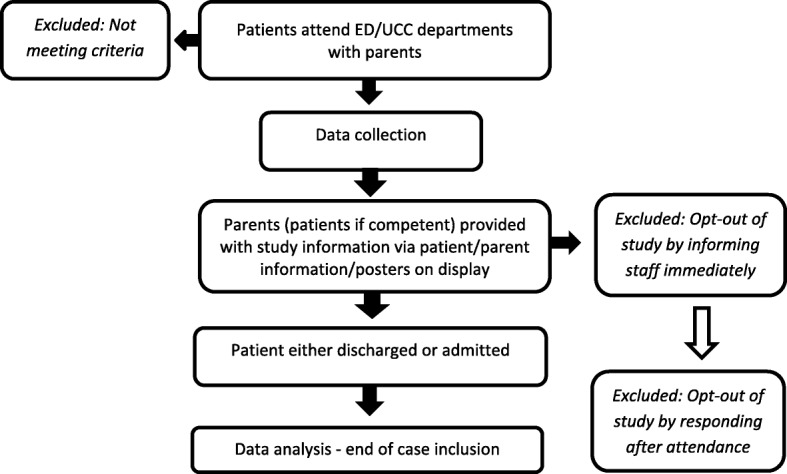
Flow of participants

The overall study flowchart is presented in Fig. 1.

### Consent and confidentiality

A number of consultation events were held with parents whose children had attended an ED in the previous 18 months. The findings were used to inform the research design with regards to our approach to parents, ethics permissions, methods of seeking consent, informing those attending the ED of the study, and study outcome measures. A study advisory group will be recruited during the study to assist the research team with recruitment, to receive and comment on reports and advise and assist with the dissemination strategy.

Patients and their families will experience no difference to their service and will suffer no additional physical or psychological risk and Parents advising the study design were clear that it would be inappropriate to add unnecessary concern at the point of triage and examination. All families will be provided with an information sheet incorporating details of how to gain additional information or to opt-out of the study. Staff in the department will be available, on request, to speak to any participant or parent regarding the study. There will be the choice to opt-out immediately or to do so later (remotely). This will be given to parents after triage and once clinical reassurance has been provided that the child is at no risk of harm. This is consistent with the decisions made by our Patient and Public Involvement (PPI) group and also by long-standing NHS research practices [17]. Formal ethical approval for this approach has been granted.

Identifiable data will be accessed and used only by members of the research team at The Pennine Acute Hospitals NHS Trust. The Data Manager will (periodically, but sometime after the clinical episode) assign study numbers to the cases before providing the data to the statistician and members of the management team.

1. The Principal Investigator, on behalf of The Pennine Acute Hospitals NHS Trust, is the custodian of the data;
2. Research participants have the right to revoke their authorization for the use of personal information;
3. Participants will not be identifiable in any future publication.

All parties involved in the study have data management strategies and associated processes for monitoring. Personal detail will remain on NHS Trust property and all study data will be anonymised.

### Data collection

#### The reference standard and its rationale

The reference standard should be the best possible method of determining the outcome and should be objective rather than subjective [18]. An objective decision on whether to admit a child or young person to inpatient care is problematic as there is no existing gold standard outcome measure for the decision to admit or discharge a child or young person from the ED. The decision to admit children and young people is a complex decision, which can vary between clinicians and hospitals.

We will define a patient as being admitted to hospital if they leave the ED to enter the hospital, (including observation and assessment unit or hospital ward), either on first presentation or with the same complaint within seven days of first presentation. This correlates well with the thoughts of the PPI group, which saw admission and discharge in such terms, more clearly than did the research group. This reference standard has been adopted after direct discussion with members of the public involved in our project and with three ED doctors who have reviewed a draft of this proposal. The decision to admit the patient will be made by a clinician (either a doctor or a nurse practitioner). They will follow existing guidelines, using usual methods of clinical judgement, and will be blinded to the PAT-POPS database and the final PAT-POPS score. Admission data from all the hospitals in the Trust will be accessed from the existing NHS Trust electronic systems.

#### PAT-POPS assessment process

The current version of PAT-POPS v1 includes age, heart rate, temperature, respiratory rate, oxygen saturation (%), requirement for supplemental oxygen, breathing, responsiveness (AVPU), nurse judgement, behaviour, chronic condition. Other screening tools for use in EDs [19] include other non-invasive variables which might improve the diagnostic accuracy of PAT-POPS. Therefore in addition to the current PAT-POPSv1, the following additional variables: arrival by ambulance; day of the week; time of the day; referral by health professional; attendance with same problem in previous week will be collected. The full list of variables to be collected is available as in Table 1. All of the potential assessment items for the PAT-POPS v2 tool will be collected from each child. Data will be collected at triage by existing clinical staff as a routine part of practice, and entered into the existing IT systems used at the study sites (Symphony and PAS). Data will be stored securely in the Symphony and PAS systems and exported to a purpose-designed database every three months. Data will be collected on all eligible patients who attend the ED/UCC thereby ensuring data is collected throughout the twenty-four hour period and throughout all days of the week.

**Table 1:** Study variables

	Variable	Variable Type	Values
Category of variable	Site ID	number	1 2 3 ….
	Participant ID	number	Start with site ID eg 100001, 100002… 200001, 200002 …
	Eligible for the study	binary	Y/N [if any of the ineligibility reasons selected this defaults to N]
	Ineligibility reason	nominal	Dead on arrival; Arrived in cardio-respiratory arrest.
	Date of arrival	date	
Patient characteristics	Gender	nominal	Male/female
	Ethnicity	nominal	African Any other Asian Background Any other Black Background Any other Ethnic Group Any other Mixed Background Any other White Background Bangladeshi British Caribbean Chinese Indian Irish Not Stated Pakistani White & Asian White & Black African White & Black Caribbean
PAT-POPs existing variables:	Date of birth	date	
	Heart rate	number	
	Temperature	number	
	Respiratory rate	number	
	Oxygen saturation (%)	number	
	Does this patient require supplemental oxygen to maintain appropriate oxygen saturation levels?	binary	Y/N
	Breathing – wheeze	binary	Y/N
	Breathing – stridor	binary	Y/N
	Breathing – audible grunt	binary	Y/N
	Breathing – tracheal tug	binary	Y/N
	Other Respiratory Distress apart from wheeze, stridor, audible grunt or tracheal tug.	binary	Y/N
	Select one of the following four (no further selections available once one has been selected)		
	1. Breathing – severe recession	binary	Y/N
	2. Breathing – moderate recession	binary	Y/N
	3. Breathing – mild recession	binary	Y/N
	4. Breathing – no recession	binary	Y/N
	Responsiveness (AVPU)	nominal	Unresponsive Responds to Pain Responds to Voice Alert
	Nurse’s judgement	nominal	Child looks unwell or High Level Concern Low level concern No concern
	Behaviour	nominal	Floppy Listless Normal for age Inappropriate Agitated
	Is there an existing co-morbidity (chronic condition)?	binary	Y/N
Additional PAT-POPs variables	Did the patient arrive by ambulance?	binary	Y/N
	Time of arrival	time	
	Has the patient been advised to attend by a medical professional?	binary	Y/N
	Has the patient visited an emergency department, urgent care centre or general practitioner with the same problem in the last 7 days?	binary	Y/N (asked at reception)
	Has the patient visited an emergency department, urgent care centre or general practitioner with the same problem in the last 7 days?		Y/N (asked at triage or nurse assessment)
Other data collection	Diagnosis	nominal	Symphony discharge diagnosis list
	Death in the ED	binary	Y/N
Admission decision	Was an admission decision made?	binary	Y/N
	If no decision, why not?	nominal	Child left ED/UCC before decision could be taken; Not known.
	Was the child admitted on this occasion?	binary	Y/N
	Was the child admitted at any point during the next 7 days?	binary	Y/N

#### Other data collection

We will also collect data on reason for attendance at the ED; diagnosis; deaths in the ED; children leaving the ED before admission decision; children’s characteristics (age, gender and ethnicity); investigated deaths and serious incidents.

### Sample size

#### Calculation of sample size of training dataset (stage 1)

The training dataset will be used to undertake clinically guided stepwise model building, using logistic regression modelling. A suitable approach to sample size estimation for building logistic regression models is to include 20 cases requiring hospital admission for each level of freedom of each variable that is being considered [20]. The variables that will be considered for inclusion in the modelling number 22 in total (see Table 1 for a full list). The POPS variables are currently calculated as categorical variables with 3 categories (0, 1 or 2), so we have assumed in the sample size estimation that all 22 variables will be measured at 2 levels. The actual cut-points of individual variables will be determined through a statistical examination of the variables with clinical opinion if the values are not valid in practice. The admission rate will vary across hospitals: we have looked at the admission rates of 3 hospitals 2010–14 and find a mean admission rate of 13%. (20 cases × 22 variables × 2 levels per variable)/0.13 = 6770 children. When undertaking the modelling, it would be helpful to consider seasonality, and examine differences between sub-groups of children, including whether they arrive with trauma or a medical complaints. To avoid overfitting the models we will therefore require a minimum of around 9000 children for the training dataset.

#### Calculation of sample size of validation dataset (stage 2)

We calculate the sample size for the validation data set using the procedure proposed by Flahault, et al. [21]. This method first estimates the expected sensitivity and specificity at the chosen cut-point of the POPS screening test. It also calculates the number of cases that are required to estimate the sensitivity and specificity to within a specified 95% confidence interval. This provides the number of cases, which then is divided by the admission rate, to estimate the total sample of children needed for the study. We conservatively estimate the expected sensitivity and specificity of PAT-POPS v3 at 0.75; we set the 95% confidence interval at 0.7 to 0.8; and assume an admission rate of 0.13 (as before). To estimate an expected sensitivity and specificity of 0.75, with a 95% confidence interval of 0.7 to 0.8, 869 admitted cases are required (using tables provided in Flahault et al.). Assuming the admission rate will be 0.13, we will need to recruit 869/0.13 = 6685 children for the validation data set.

The minimum sample size needed to do both analyses is 16,000 children.

### Data collection

The data used to calculate PAT-POPS v2 will be collected for all children and young people attending the ED and UCC during the 12 month period February 2018 to January 2019, at the three hospitals.

Our estimated minimum sample size required for the analysis is 16,000 children). It is likely that the 12 month period will allow for over-recruitment which is necessary for the following reasons.

- The need to collect data for a full year to capture seasonal variation in childhood illness and injury.
- Intermittent data collection would not help implementation of the tool.
- Intermittent data collection would require us to employ specific staff for the project, which would be more costly.
- Information technology failure at all, or one of the sites, is not in the control of the study team.

A third of the patients (from one of the hospitals) will be assigned to a training set and the remainder, from the other two hospitals, to a validation dataset. The justification for this approach is that we will develop the PAT-POPS tool using one of the EDs and then validate it using a different ED and UCC.

### Statistical methods and analysis

A preliminary statistical examination will be undertaken in Spring 2018 of all data collected up to that time point. The purpose of this is to estimate the final sample size, assess the suitability of variables for analysis, explore collinearity between variables, make preliminary decisions on the list of candidate variables and how they will be categorised or transformed and check any differences between the three sites.

The final analysis will be undertaken after all the data has been collected. The preliminary analysis will be repeated, to confirm the list of candidate variables and identify any changes since the earlier analysis.

### Stage 1 – Training dataset – Developing a prognostic model

Children from one hospital site will be utilised for the stage 1 analysis.

#### Refine the PAT-POPS tool

The aim is to identify which items to include in the revised PAT-POPS v2 tool. We will achieve this by developing logistic regression models with hospital admission as the outcome and include all candidate variables (both the subjective and objective components of the PAT-POPS tool). See Table 1 for the list of variables to be collected. Our model building approach will be stepwise and decisions on item inclusion will be clinically guided. We will start by examining the relationship between each model variable and the outcome, to assess for the degree of linearity and to identify suitable cut-points for continuous variables. We will then build logistic regression models, guided by clinical opinion from our research team. We will compare the suitability of models using AIC. A summary of the demographic characteristics, health status and diagnostic characteristics for this patient population will be reported. Responses to all individual PAT-POPS items will be presented. The frequency of the reference standard will be reported. The number of missed patients will be reported as will any drop-outs during the study and the reason for any drop out. Multiple imputation will be considered if the preliminary analysis indicates it is a suitable approach with the available data. We will assess how well the model performs by reporting model fit (Brier’s score), calibration and discrimination (C-statistic, equivalent to AUROC). We are not planning any internal validation because the expected large sample size makes it unlikely that we will have a problem with over-fitting or optimism.

The output of Stage 1 will be a prognostic model which can identify the variables to include in a PAT-POPS clinical decision tool, and the relative weight of those variables in predicting hospital admission and discharge.

### Stage 2 – Training dataset - Conversion of the model to a clinically useful tool

We will use the parameters from the multivariable model developed in Stage 1 to assign integer points to the level of each risk factor, and produce a reference table for a clinically useful score.

#### Sensitivity and specificity – Identify cut points

We will calculate the sensitivity, specificity, positive and negative likelihood ratios of PAT-POPS v2 (index test) to predict admission (reference test), at all possible cut points of PAT-POPS [20], with 95% confidence intervals. A cross tabulation of the results of the index test by the results of the reference test will be reported, including indeterminate and missing results.

#### Consensus meeting – Agree PAT-POPS cut points

We will organise a meeting to examine the statistical data, and agree which cut points of PAT-POPS are most suitable to predict (i) safe admission decision and (ii) safe discharge decision, including consideration of what weight to give to sensitivity and specificity in making the decision. We will invite all of our research team, plus 2 independent paediatric ED clinicians, 2 independent methodologists and 2 members of the public involvement group. Prior to the meeting, we will hold a separate meeting, to brief the public involvement group members and ensure that they understand the basics of the statistical methods involved and are clear in what is expected of them – provision of a service-user view rather than technical expertise. The members of the public will be supported in the meeting by Dr. Livesley (who will also lead on their training programme and support by the whole team).

### Stage 3 – External validation dataset

All children attending the other two hospitals will be utilised for the stage 2 analysis. We will assess the usefulness of the PAT-POPS tool by calculating the sensitivity, specificity, positive and negative likelihood ratios of PAT-POPS v2, at the chosen cut-points, to predict admission and discharge. We will compare the sensitivity and specificity of PAT-POPS v1 and PAT-POPS v2 to predict admission or discharge using the DeLong method to compare ROC curves [22] and reporting the sensitivity and specificity at the chosen cut-points.

We will compare the sensitivity and specificity of PAT-POPS v2 to predict admission for separate groups of children and young people with illness or trauma, using the DeLong method to compare ROC curves [23] and reporting the sensitivity and specificity at the chosen cut-points. We will report the incidence of investigated deaths and serious incidents and report whether or not these would have been picked up by the PAT-POPS tool. All analysis will be undertaken using STATA 15 or later [24]. A detailed Statistical Analysis Plan will be written prior to the end of data collection. It will be drafted by Sarah Cotterill and approved by the management group.

### Study monitoring and risk assessment

Quality control and quality assurance are in place to ensure that all elements of the PAT-POPS study are performed in compliance with applicable regulatory requirements. These are undertaken by the Steering Group (SG).

### Study approval

PAT-POPS was given favourable opinion by the West Midlands (Black Country) Research Ethics Committee on the 20th of December 2017. PAT-POPS received HRA approval on the 22nd of December 2017. The sponsor confirmed capacity and capability to deliver the study on the 2nd of January 2018.

## Discussion

### Expected impact of the research

Leicester POPS, and its derivative PAT-POPS, have demonstrated an ease of uptake and transferability. Once a validated, and more accurate, model is developed the applications for the wider NHS and patient benefit could be substantial.

1. The PAT-POPS tool is easily implemented into other urgent and emergency care settings throughout the NHS. The tool requires no additional infrastructure as its components are based on standard assessments already occurring. An education package already exists and the learning from this study will enable a toolkit to be developed which can be disseminated easily to organisations and/or commissioning groups.
2. PAT-POPS will allow commissioners to assess the relative acuity of presentations of children and young people between urgent and emergency care centres in their service areas. This will allow enhanced workforce planning and service delivery models to be developed.
3. PAT-POPS can be used to reassure families in an objective manner that they are being managed in an environment suitable for their child’s clinical condition. This will improve the families’ experience of care.

## Abbreviations

AIC: Akaike Information Criterion
AVPU: Alert, Voice, Pain, Unresponsive
CEMACH: The Confidential Enquiry into Maternal and Childhood Health
ED: Emergency Department
HRA: Health Research Authority
NHS: National Health Service
NIHR RfPB: National Institute for Health Research Research for Patient Benefit programme
PAS: Patient Administration System
PAT-POPS: The Pennine Acute Hospitals NHS Trust Paediatric Observation Priority Score
PPI: Patient and Public Involvement
RDS: Research Design Service
REC: Research Ethics Committee
ROC: Receiver Operating Characteristic
SG: Steering Group
UCC: Urgent Care Centre
